# A Deep Learning Model for Detecting the Eyes Receiving Glaucoma Medications Using Anterior Segment Images

**DOI:** 10.1167/tvst.14.8.28

**Published:** 2025-08-20

**Authors:** Shogo Arimura, Ryohei Komori, Kentaro Iwasaki, Marie Suzuki, Yusuke Orii, Masaru Inatani

**Affiliations:** 1 Department of Ophthalmology, Faculty of Medical Sciences, University of Fukui, Matsuoka, Eiheiji, Yoshida, Fukui, Japan

**Keywords:** deep learning, glaucoma medications, anterior segment images, ocular surface, saliency mapping

## Abstract

**Purpose:**

We aimed to investigate whether a deep learning model can detect eyes receiving glaucoma medications from anterior segment images and to visualize the anatomical areas prioritized during classification.

**Methods:**

The training dataset was comprised of 20,000 augmented images of eyes receiving or not receiving glaucoma medications. The test dataset was comprised of 100 images each of eyes receiving and not receiving glaucoma medications. Diagnostic performance of the model was evaluated using the area under the receiver operating characteristic curve (AROC) and compared with human recognition. Subgroup analyses were performed based on conjunctival hyperemia, prostaglandin analog use, and illumination conditions. Gradient-Weighted Class Activation Mapping (Grad-CAM) was applied to explore anatomical areas prioritized by the model.

**Results:**

The deep learning model detected the eyes receiving glaucoma medications with significantly higher accuracy than human recognition (AROC, 0.90 vs. 0.75; *P* < 0.01). No significant AROC differences were observed in the presence or absence of conjunctival hyperemia, prostaglandin analog use, or under varying illumination. Grad-CAM analysis revealed the periocular area was significantly more frequently highlighted in eyes receiving glaucoma medication than in those not receiving medication (*P* < 0.01).

**Conclusions:**

The deep learning model objectively detected glaucoma medication use based on anterior segment images. Saliency mapping suggests that the model can identify subtle periocular changes induced by treatment.

**Translational Relevance:**

The deep learning model will contribute to assessing the severity of side-effects of glaucoma medications and facilitate the development of eye drops with improved tolerability.

## Introduction

Glaucoma is a leading cause of moderate to severe vision impairment worldwide.^1^^,^^2^ Glaucoma medication as topical eye-drop therapy is initiated to control intraocular pressure in patients with glaucoma. Glaucoma medications are indispensable as an initial treatment; however, they are associated with risks of side effects. The common side effects include ocular surface complications,^3^^–^^5^ such as discomfort, tears, conjunctival hyperemia, and eyelid troubles. Prostaglandin-associated periorbitopathy (PAP)^6^^,^^7^ is a well-known side effect of prostaglandin analog eye drops for glaucoma patients and includes eyelash elongation, eyelid and iris pigmentation, and eyelid sulcus deepening. Due to the side effects of glaucoma medications, clinically observable changes on the ocular surface including conjunctival hyperemia and PAP may occur, and no objective devices and algorithms have been developed to detect these changes.

The accuracy of image recognition using deep learning machine models is remarkably high. High precision in gender and sex identification from fundus or optical coherence tomography images using a deep learning model has been indicated in several studies.^8^^–^^10^ Regarding glaucoma, its presence and severity have been accurately detected from fundus and optical coherence tomography images in previous studies.^11^^–^^13^ Furthermore, machine learning models perform excellently in assessing the disease based on anterior segment images. Studies using machine learning models to detect eye diseases with high accuracy at the anterior segment level, such as corneal diseases,^14^^,^^15^ conjunctivitis,^16^ and the presence of pterygium,^17^ have been conducted. Glaucoma medications may cause visually apparent changes in the anterior segment of the eyes.

Therefore, we considered that a deep learning model could mechanically assess the complications associated with glaucoma medications. Here, we aimed to explore whether a deep learning algorithm can detect the eyes receiving glaucoma medications based on anterior segment images. Furthermore, we employed Gradient-Weighted Class Activation Mapping (Grad-CAM) to visualize the areas of the image the model focused on, in order to gain insight into the morphological features contributing to the predictions of the model.

## Methods

This study was approved by the institutional review board of Fukui University Hospital, Fukui, Japan, which waived the requirement for informed consent because of the retrospective nature of this study. All methods adhered to the tenets of the Declaration of Helsinki for research involving human subjects, and this study was conducted in accordance with the regulations of the Health Insurance Portability and Accountability Act.

### Training and Test Dataset

**Figure 1.**
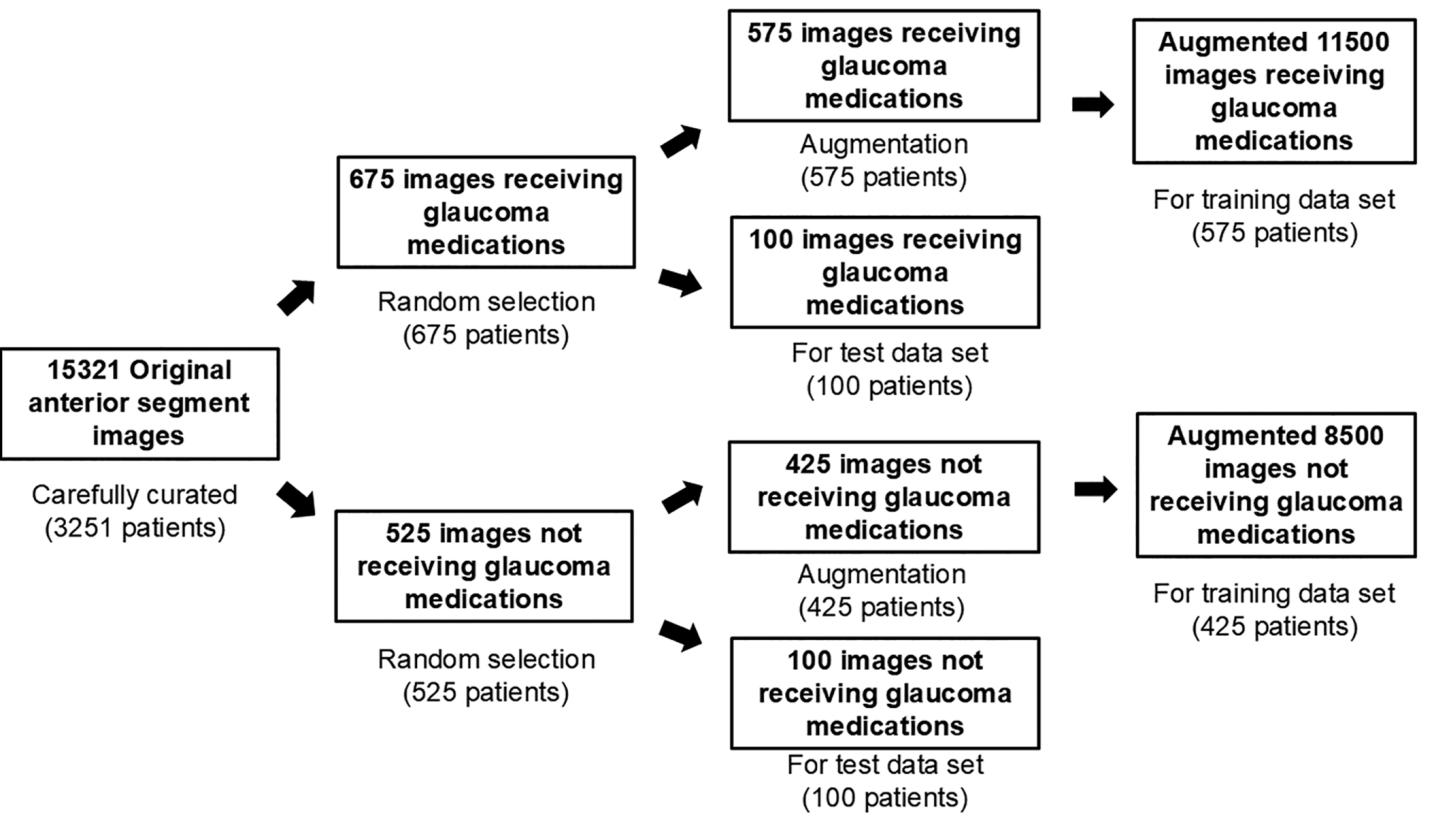
Selection of anterior segment images for training and test datasets. The flowchart depicts the allocation of images into the training and test datasets.

We initially extracted 15,321 anterior segment images from clinical records between January 2015 and December 2023, representing 3251 unique patients. After quality-based curation to exclude images that were blurry, dark, overexposed, or partially occluded, a high-quality subset was selected, including 675 images from 675 patients who had been receiving glaucoma medications and 525 images from 525 patients who had not. From this curated dataset, the training dataset prior to data augmentation was constructed using 575 images from 575 patients in the receiving glaucoma medication group and 425 images from 425 patients in the not receiving glaucoma medication group. The training dataset after data augmentation was constructed of a total of 20,000 images. Data augmentation of images was performed using left-right flipping, rotation, changes in hue and color tone, and saturation on OpenCV 4.5.1. The test dataset was comprised of 100 images from 100 unique patients in each group (200 total), with each patient contributing only one image. To prevent data leakage, we strictly ensured that no patient appeared in both training and test datasets. Figure 1 has been provided to visually clarify the above patient-level selection and data splitting process. The anterior segment images were captured using a camera (THD-23FHD; Ikegami Tsushinki Co., Ltd., Tokyo, Japan) integrated with a slit-lamp microscope during the patient's examination. The slit-lamp light, either slit or diffuse, was used. The ocular surface was entirely illuminated by the external lighting attached to the slit lamp. The resolution of images captured in full high definition (HD) was approximately 2.07 million pixels, with a resolution of 1920 × 1080. The room illuminance was 0 to 50 lux during the slit-lamp examination. The anterior segment images included the central cornea, bulbar conjunctiva, limbal area, and periocular area. The field of view was approximately 30° to 45°, which is the standard setting for anterior segment photography using slit-lamp mounted cameras. We resized all images to a dimension of 224 × 224 pixels when loading images into the machine learning model.

### Deep Learning Model

We performed transfer learning using Visual Geometry Group 16 (VGG16).^18^ VGG16 is a convolutional neural network model consisting of 16 layers trained on a large-scale image dataset referred to as ImageNet. This 16-layer convolutional neural network model includes an input layer with a color channel size of 224 × 224 and an output layer for 1000-class classification. In the present study, we modified the last layer of VGG16 to classify images into two categories. In addition, the weights of the first 15 layers were fixed to reduce processing time. Fixing the weights refers to the process where part of the weights of a pretrained model are not updated. In particular, fixing the weights of each layer of VGG16 ensures that these weights are not updated during the training of the new dataset. The number of epochs was set to 40, and the batch size was 16 in the deep learning model. Categorical cross-entropy was applied, yielding the output of class probabilities. Optimization was performed using the Adam algorithm, which combines momentum and adaptive learning rates. The general design framework of the model is provided in Supplementary Table S1.

### Performance of the Deep Learning Model

The performance of the developed model was assessed using the area under the receiver operating characteristic curve (AROC), sensitivity, and specificity. In addition, the model was evaluated separately per section; Section 1 was the primary outcome in this study, and Sections 2, 3, and 4 were the secondary outcomes. We further conducted a stratified analysis of model performance based on the number of glaucoma medications used, as measured by the AROC.

**Figure 2.**
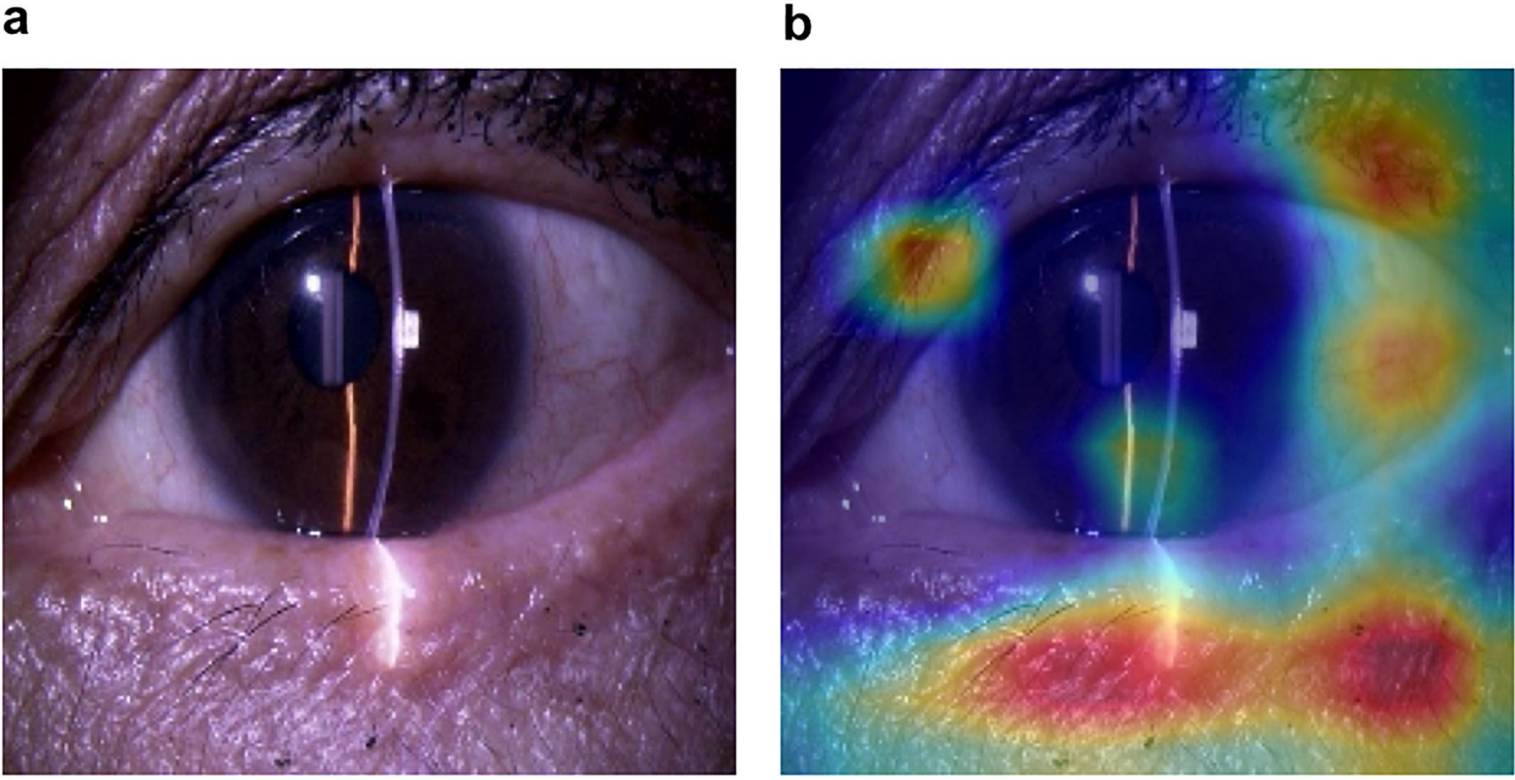
(**a**, **b**) Saliency map analysis using Grad-CAM, with representative images of the original (**a**) and the saliency map (**b**).

To explore which anatomical areas the deep learning model prioritized in its classification decisions, we conducted a saliency map analysis using Grad-CAM (Fig. 2). Grad-CAM heatmaps were generated for all test images, and the highlighted areas were manually annotated and categorized into three anatomical regions: (1) central anterior segment including the cornea and iris; (2) perilimbal area including the conjunctiva, sclera, and associated vasculature; and (3) periocular area, primarily involving the upper and lower eyelids, and eyelash.

For each image, the region most prominently highlighted by Grad-CAM was identified. Frequencies of attention in each region were compared between eyes with the presence or absence of glaucoma medication using χ^2^ tests. In addition, for region 3, statistical analysis was conducted between eyes treated with prostaglandin analogs and eyes not receiving glaucoma medications, considering the potential influence of PAP.

#### Section 1: Deep Learning Model Compared With Human Recognition

The AROC, sensitivity, and specificity of the deep learning model were compared with those of human recognition. We investigated whether humans could recognize the eyes receiving glaucoma medications based on anterior segment images, which were the same images in the test dataset of the deep learning model. The AROC of human recognition was calculated as the average of the AROCs of five glaucoma specialists. We also focused on both false-negative and false-positive images identified using the deep learning algorithm. A false negative was defined as a case in which the patient actually used glaucoma medications but the model failed to identify it. A false positive referred to a case in which the model incorrectly identified the use of glaucoma medication in a non-treated eye. In addition, we evaluated whether these misclassified images overlapped with those misclassified by human graders. For each of the false-negative and false-positive cases identified by the model, we assessed whether human recognition also misclassified the same image. We then conducted Fisher's exact test to determine whether the overlap between model and human misclassifications was statistically significant.

#### Section 2: Conjunctival Hyperemia

We investigated whether the ability of the deep learning model changes depending on the presence or absence of conjunctival hyperemia. We compared the AROC of the model (same as in Section 1) with the AROCs of the presence and absence images of conjunctival hyperemia, respectively. In addition, we compared the AROCs between the presence and absence of hyperemia groups. The presence of hyperemia was determined according to Japanese guidelines for allergic conjunctival diseases.^19^ A score of 0 indicated no conjunctival hyperemia, whereas other scores indicated the presence of conjunctival hyperemia. The presence or absence of hyperemia was labeled by two board-certified ophthalmologists (SA, MI) based on slit-lamp photographs. Both graders independently assessed the images, and any discrepancies were resolved through discussion and consensus.

#### Section 3: Prostaglandin Analog Eye Drops

We investigated whether the ability of the model changes depending on the presence or absence of prostaglandin analog eye drops. Prostaglandin analog eye drops are the first treatment option for glaucoma, and their side effects, including PAP, are considered to have the greatest impact on changes to the ocular surface. In this section, we compared the AROC of the deep learning model (same as in Section 1) with the AROCs obtained from images of eyes using prostaglandin analogs and those not using prostaglandin analogs. Additionally, we compared the AROCs between the eyes with prostaglandin analog and without prostaglandin analog groups.

#### Section 4: Illumination Conditions

To evaluate the potential influence of image acquisition conditions on model performance, we further stratified the test images based on illumination type. Anterior segment images were categorized into two groups: those obtained under slit illumination and those under diffuse illumination. The performance metrics, including AROC, accuracy, sensitivity, and specificity, were compared between the two groups to assess any variation attributable to lighting conditions.

### Inclusion and Exclusion Criteria

This study included patients who had been using glaucoma medications for >6 months. Eyes with conjunctivitis and blepharitis were intentionally included to reflect real-world clinical variability. The exclusion criteria were as follows: patients who had undergone any ocular surgery including cataract extraction or minimally invasive glaucoma surgery within 6 months prior to the date of photography, to avoid bias from transient postoperative ocular surface changes; patients with a history of glaucoma filtering surgeries, such as trabeculectomy and express shunt insertion or long tube shunt surgeries; patients who had undergone surgical interventions that caused an invasion of the conjunctiva, including procedures related to eyelids, pterygium, and tumor removal; and patients with severe ocular pathology such as corneal ulcers, active or previous keratitis, ocular surface tumors, and severe dry eye syndrome.

### Statistical Analysis

Each patient contributed images to only one group, and no patient was included in both groups. Accordingly, all statistical analyses were performed assuming group independence. All statistical analyses were performed using the statistical programming language Python 2.7.9 (Python Software Foundation, Beaverton, OR) and SPSS Statistics 27.0 (IBM, Chicago, IL). Pearson's χ^2^ or Fisher's exact test was performed for categorical data. Unpaired *t*-tests were performed for normally distributed continuous data. Statistical significance was set at *P* < 0.05. AROCs were compared using DeLong's method.^20^ We set the significance level to 0.05 and power to 0.9 in the sample size calculation. Based on a preliminary small-scale study, it was assumed that the AROC of the deep learning model was 0.9 and that of human recognition was 0.7.

## Results

Table 1 presents the baseline data of patients in the test dataset. A significantly higher number of glaucoma medications and prevalence of conjunctival hyperemia were observed among images of eyes receiving glaucoma medication compared with images of eyes not receiving glaucoma medication (*P* < 0.01). Supplementary Table S2 presents the baseline data of patients in the training dataset. Significantly higher numbers of glaucoma medications, prevalence of conjunctival hyperemia, blepharitis, and superficial punctual keratitis were observed among images of eyes receiving glaucoma medication than among images of eyes not receiving glaucoma medication (*P* < 0.01). Supplementary Tables S3 presents the type and rate of eye drops in images of eyes receiving glaucoma medications. We further stratified model performance by the number of glaucoma medications used. The AROCs were 0.86 for patients using one medication (*n* = 15), 0.87 for two medications (*n* = 16), and 0.93 for those using three or more medications (*n* = 56).

**Table 1.** Baseline Characteristics in The Test Dataset

	Receiving Glaucoma Medication	Not Receiving Glaucoma Medication	*P*
Number	100	100	—
Age (y), mean ± SD	70.0 ± 10.8	68.9 ± 11.0	0.48
Sex, *n* (%)			0.48
Male	51 (51.0)	46 (46.0)	
Female	49 (49.0)	54 (54.0)	
Eye, *n* (%)			0.53
Right	50 (50.0)	58 (58.0)	
Left	50 (50.0)	42 (42.0)	
Glaucoma medications (*n*), mean ± SD	2.8 ± 1.4	0	<0.01^a^
Lens status, *n* (%)			0.39
Phakic	57 (57.0)	63 (63.0)	
Intraocular	43 (43.0)	37 (37.0)	
Conjunctival hyperemia, *n* (%)	64 (64.0)	31 (31.0)	<0.01^a^
Blepharitis, *n* (%)	20 (20.0)	11 (11.0)	0.12
Superficial punctual keratitis, *n* (%)	17 (17.0)	9 (9.0)	0.14

a Statistically significant (*P* < 0.05). Unpaired *t-*tests, Pearson's χ^2^ test, and Fisher's exact test were used for the analyses.

Grad-CAM–based saliency analysis revealed differential model attention depending on medication status (Table 2). Among eyes receiving glaucoma medications, the most frequently highlighted region was the periocular area (region 3), observed in 75 out of 100 cases (75.0%), compared to 47 out of 100 cases (47.0%) in eyes not receiving glaucoma medication. This difference was statistically significant (*P* < 0.01). Among the 75 cases in which the Grad-CAM analysis highlighted region 3, 74 cases were using prostaglandin analogs, yielding a concordance rate of 98.7%. Additionally, we conducted a statistical comparison between the eyes of prostaglandin analog users and those not receiving glaucoma medication groups within region 3, which revealed a significant difference (*P* < 0.01). In contrast, there were no significant differences between groups in the central anterior segment or the perilimbal region.

**Table 2.** Grad-CAM Saliency Map Analysis

Region	Receiving Glaucoma Medication	Not Receiving Glaucoma Medication	*P*
1	35.0%	45.0%	0.19
2	55.0%	56.0%	1.00
3	75.0%	47.0%	< 0.01^a^

Region 1 was comprised of the central anterior segment, including the cornea and iris; region 2, perilimbal area including the conjunctiva, sclera, and associated vasculature; and region 3, periocular area, primarily involving the upper and lower eyelids.

a Statistically significant (*P* < 0.05). Fisher's exact test was used for the analyses.

### Section 1: Deep Learning Model Compared With Human Recognition

**Figure 3.**
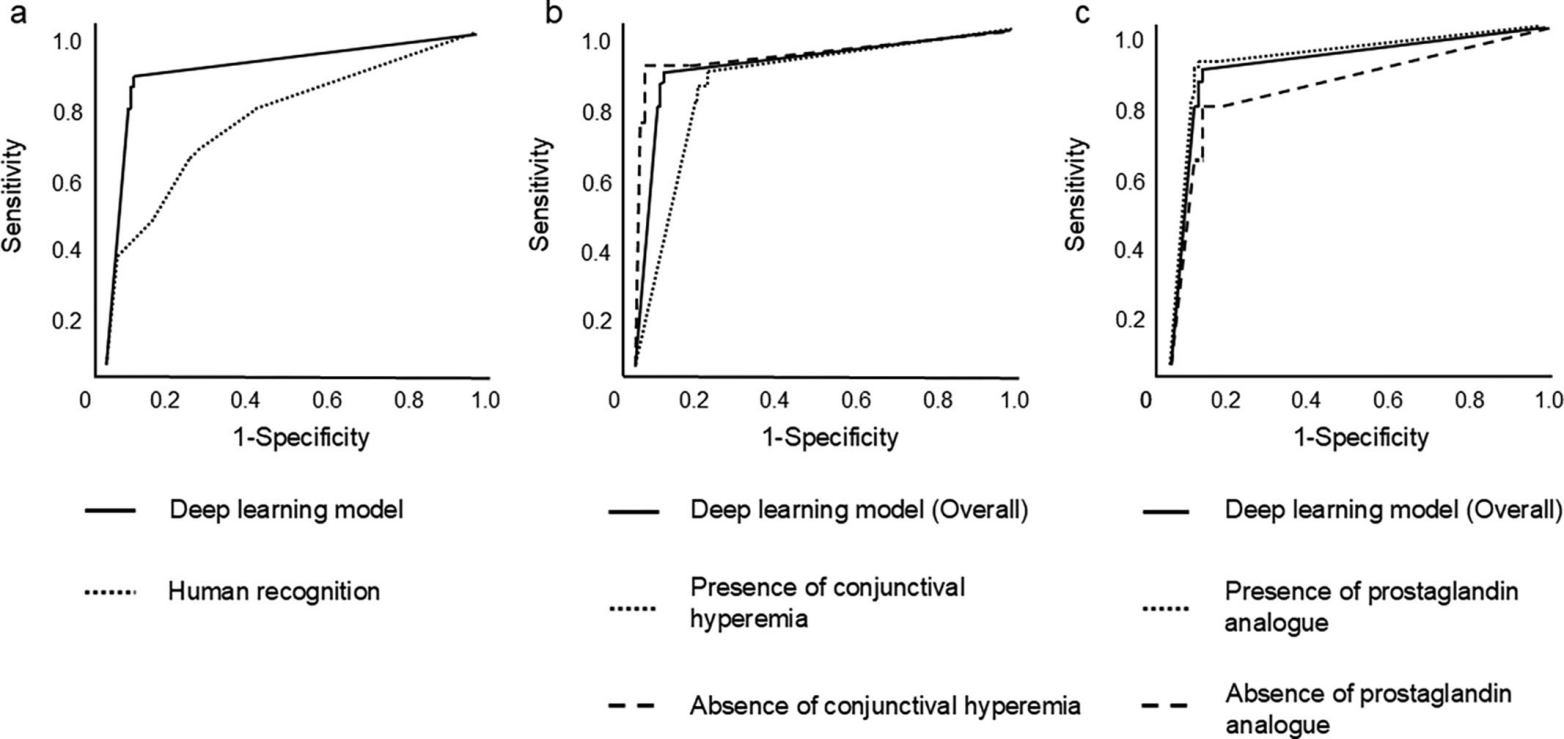
Comparison of AROCs in Sections 1, 2, and 3. (**a**) Comparison of AROCs between the machine learning model and human recognition. The deep learning model had a higher AROC of 0.90 for classification, whereas the AROC of human recognition was 0.75 (*P* < 0.01). (**b**) Comparison between overall AROC and AROC in the presence or absence of conjunctival hyperemia. There were no significant differences between the overall AROC and AROC in the presence or absence of conjunctival hyperemia (*P* = 0.30 and *P* = 0.50, respectively). The difference in AROCS between the presence and absence of conjunctival hyperemic groups was not statistically significant (*P* = 0.10). (**c**) Comparison between overall AROC and AROC in the presence or absence of prostaglandin analog eye drops. There were no significant differences between the overall AROC and AROC in the presence or absence of prostaglandin analog eye drops (*P* = 0.79 and *P* = 0.42, respectively). The difference in AROCs between the presence and absence of prostaglandin was not statistically significant (*P* = 0.34).

Figure 3a shows the AROCs of the deep learning model and human recognition. The deep learning model had a significantly higher AROC than did human recognition (0.90 vs. 0.75; *P* < 0.01). The accuracy, sensitivity, and specificity of the deep learning model were 88.5%, 85.0%, and 92.0%, respectively, whereas those of human recognition were 65%, 48%, and 83%, respectively. As a result of the machine learning model classification, 13 out of 100 images were identified as false negatives, and 8 out of 100 images were identified as false positives. In addition, we evaluated the overlap of misclassified cases between the model and glaucoma specialists. Among the 13 false-negative cases identified by the model, 10 cases (76.9%) were also misclassified by humans. Of the eight false-positive cases, six cases (75.0%) were misclassified by humans. Fisher's exact test revealed no statistically significant difference in misclassification overlap between the model and human graders (*P* = 1.0). Supplementary Figures S1 and S2 show the false-negative images and false-positive images, respectively, and Supplementary Table S4 presents the glaucoma medication contents in each false-negative case.

### Section 2: Conjunctival Hyperemia

Supplementary Table S5 presents the baseline data regarding the presence and absence of conjunctival hyperemia in Section 2. The AROC in the presence of conjunctival hyperemia was 85%, whereas that in the absence of conjunctival hyperemia was 93%. No significant differences were noted between the overall AROC and AROC in the presence or absence of conjunctival hyperemia (*P* = 0.30 and *P* = 0.50, respectively). Additionally, we compared the AROCs between the presence and absence of conjunctival hyperemic groups. The difference between AROCs was not statistically significant (*P* = 0.10) (Fig. 3b). The accuracy, sensitivity, and specificity of the model in the presence of conjunctival hyperemia were 82.1%, 82.8%, and 80.6%, respectively, whereas those in the absence of conjunctival hyperemia were 94.3%, 88.9%, and 97.1%, respectively.

### Section 3: Prostaglandin Analog Eye Drops

Supplementary Table S6 presents the baseline data regarding the presence and absence of prostaglandin analog eye drops in Section 3. The AROC of the deep learning model in the presence of prostaglandin analog eye drops was 0.91, whereas that in the absence of prostaglandin analog eye drops was 0.84. No significant differences were noted between the overall AROC and AROCs in the presence or absence of prostaglandin analog eye drops (*P* = 0.79 and *P* = 0.42, respectively) The difference of AROCs between the presence and absence of prostaglandin analog was not statistically significant (*P* = 0.34) (Fig. 3c). The accuracy, sensitivity, and specificity of the model in the presence of prostaglandin analog eye drops were 89.0%, 86.0%, and 92.0%, respectively, whereas those in the absence of prostaglandin analog eye drops were 89.4%, 69.2%, and 92.0%, respectively.

### Comparison of Illumination Conditions

We compared the performance of the deep learning model on images acquired under slit illumination and diffuse illumination. The AROC was 0.90 for slit illumination and 0.89 for diffuse illumination, with no statistically significant difference (*P* = 0.88). For slit illumination, the accuracy, sensitivity, and specificity were 90%, 84%, and 95%, respectively. For diffuse illumination, these values were 88%, 87%, and 90%, respectively. These findings suggest that the model maintained robust performance across different lighting conditions.

## Discussion

The present study is the first to show that the deep learning model detects the eyes receiving glaucoma medications based on anterior segment images. The model exhibited superior performance compared with human recognition. We initially hypothesized that clinically observable changes on the ocular surface, including conjunctival hyperemia and PAP, due to glaucoma medications would result in significant differences in the AROC, but there were no significant differences in the AROC of the model in the presence or absence of conjunctival hyperemia and the use of prostaglandin analog eye drops. However, saliency map analysis provided further insight into the behavior of the model. Grad-CAM visualizations showed that the model significantly more often focused on the periocular area in eyes receiving glaucoma medications. Furthermore, a statistically significant difference was observed between eyes receiving prostaglandin analog eye drops and those not receiving glaucoma medication. This region may reflect subtle medication-induced changes, such as those associated with PAP, which are not limited to conjunctival hyperemia. Our findings suggest that the model may be capturing drug-induced anatomical alterations that are not readily recognized or quantified by clinicians. The deep learning model bridges this critical gap by offering a non-invasive and reproducible tool capable of detecting early anatomical changes in the ocular surface and periocular tissues induced by medication. This may enable clinicians to more precisely assess the severity of side effects and make better-informed decisions regarding medication selection or switching and may ultimately facilitate more personalized glaucoma management, as well as the development and evaluation of next-generation eye drops with improved tolerability.

In the analysis of anterior segment images, deep learning models demonstrate high detection rates and a high ability to distinguish diseases.^21^^–^^23^ Wu et al.^24^ classified cataracts and assessed their severity using anterior segment images. They reported a high accuracy of 98.9% to 99.9% for the classification of cataracts and 91.9% to 100% for severity assessment. Li et al.^13^ and Gu et al.^14^ developed a program using anterior segment images to distinguish between eyes with keratitis and normal eyes or those with other conditions. In their studies, the sensitivity and specificity of the program for classification were 97.7% and 98.2%, respectively. In the present study, the deep learning model demonstrated a high detection accuracy for anterior segment changes, consistent with previous findings. Additionally, our additional stratified analysis based on illumination type demonstrated no substantial performance difference between slit and diffuse lighting conditions. The AROC, accuracy, sensitivity, and specificity remained comparable between both modalities. This suggests that the deep learning model is resilient to variations in lighting setups typically encountered in clinical practice, enhancing its generalizability and potential for real-world deployment. Furthermore, we found that the deep learning model can detect subtle alterations in the anterior segment of the eyes, such as whether glaucoma treatment medications have been administered. The model may rely on subtle and possibly subclinical features such as microtextural changes in the conjunctiva or cornea, alterations in the limbal or eyelid margin, or fine shifts in reflectivity or vessel tortuosity.^25^ These cues may not be visible to human graders but could serve as early markers of chronic medication use.

Deep learning models demonstrate high performance in evaluating and distinguishing conjunctival hyperemia. In a previous study, a model was developed to quantify the vascular occupancy rate of conjunctival congestion, and its usefulness in assessing severity was reported.^26^ Another study showed that a deep learning model performed excellently in grading the severity of bulbar conjunctival hyperemia, achieving a high AROC of 0.98.^27^ Prostaglandin analog eye drops are known to cause a variety of alterations on the ocular surface, including conjunctival hyperemia,^28^^,^^29^ tear film instability,^4^^,^^30^ reductions in corneal hysteresis and thickness,^31^^,^^32^ and PAP. These changes can result in cosmetic concerns^33^ and may impose a burden on patients. In the present study, subgroup analyses revealed no statistically significant differences in AROC between the presence and absence of conjunctival hyperemia (*P* = 0.10) or between the presence and absence of prostaglandin analog use (*P* = 0.34). However, statistical non-significance does not necessarily imply clinical irrelevance. The subgroup without hyperemia demonstrated higher specificity (97.1%) compared to the group with hyperemia (80.6%). Similarly, sensitivity was higher in the subgroup using prostaglandin analogs (86.0%) than in the non-use subgroup (69.2%). These findings suggest that the model may have partially relied on visual cues related to ocular surface changes, such as vascular congestion or periocular morphological alterations induced by medication. Differences in the number of glaucoma medications, prostaglandin analog use, and the degree of conjunctival hyperemia may have influenced the performance of the model. Specifically, the AROC of the model increased with the number of medications, possibly reflecting more pronounced ocular surface alterations in patients with greater medication burden. Furthermore, the frequency and severity of hyperemia differed between prostaglandin analog users and non-users. Among prostaglandin analog users, hyperemia was observed in 69.0% of cases. In contrast, among non-users, only 30.8% exhibited hyperemia, and none was classified as grade 3. These findings are consistent with the known association between prostaglandin analog and ocular surface inflammation and suggest that the model may partially rely on visual features related to these drug-induced changes. Given the limited sample sizes in certain subgroups (for example, only 13 images in the prostaglandin analog non-use group), it is possible that the power to detect statistically significant differences was insufficient. Nevertheless, the observed trends may reflect underlying biological or morphological features that the deep learning model is capturing. It is therefore important to recognize that relying solely on *P* values may obscure subtle yet clinically meaningful patterns. These observations further support the conclusion of this study, which is that deep learning models may contribute to the evaluation of medication-related ocular surface changes and assist in the development of glaucoma therapies with fewer side effects.

To further explore whether the model and glaucoma specialists rely on different visual cues, we examined the overlap in misclassified cases between the deep learning model and human graders. Among the 100 test images, the model yielded 13 false negatives and eight false positives. A substantial proportion of these misclassified cases were also incorrectly identified by the human graders, indicating that both the model and clinicians tend to struggle with similar challenging images. Fisher's exact test revealed no statistically significant difference in the distribution of misclassified images between the model and the human experts (*P* = 1.0). This suggests that, although both the model and clinicians frequently misclassify the same images due to subtle or ambiguous ocular surface features, they may rely on different visual patterns during classification. These findings support the notion that deep learning algorithms may detect features in medical images that are not readily perceived by human observers in clinical settings. It is also possible that some of the false-negative cases, which are listed in Supplementary Table S4, involved glaucoma medications with minimal visible effects on the ocular surface, making detection difficult even for a high-performing model.

This study has some limitations. First, the anterior segment images used in the present study were obtained from a single facility. Images captured using different cameras and in varied environments across multiple facilities might have led to improvements in the accuracy of the model. Second, there were many cases of glaucoma among the images of eyes receiving glaucoma medications, and there were mostly cases without glaucoma among images of eyes not receiving glaucoma medications. The deep learning model potentially detected the glaucoma based on anterior segment images. Third, in the absence of prostaglandin analog group, the number of glaucoma medication receiving eyes was small, which may limit the interpretability of the performance metrics of this subgroup. A fourth limitation of this study is the variability in the visibility of periocular structures, such as the eyelids and eyelashes, across the image dataset. Although these structures were partially visible in all images, the image selection protocol was not designed to consistently include the entire periocular region. As a result, subtle morphological changes associated with PAP may not have been uniformly presented to the model. This inconsistency may have introduced unmeasured confounding. Future studies may benefit from adopting standardized imaging protocols that ensure complete visualization of the periocular area when evaluating the side effects of topical glaucoma medications. Fifth, there are various types of glaucoma medications, and patients commonly use multiple medications rather than a single medication. In this study, we did not analyze the performance of the model based on the number or type of medications. Sixth, we did not perform verification on transfer learning with other neural networks. Transfer learning based on other neural networks, such as Inception, ResNet, and MobileNet, can cause a difference in results. Seventh, the test dataset consisted of one image per patient from 200 individuals, representing only a small fraction of the overall dataset; therefore, the results may be influenced by sampling variability. Although data leakage was strictly avoided by maintaining patient-level separation between the training and test sets, future studies will have to incorporate larger and more diverse datasets to enhance the generalizability of the model. Finally, our study did not directly evaluate adherence in the patient population. Further investigation is warranted to explore the relationship between image analysis using artificial intelligence and medication adherence.

In conclusion, this study provides the first evidence, to our knowledge, that a deep learning model can detect the use of glaucoma medications from anterior segment photographs with high accuracy, surpassing that of human specialists. Grad-CAM visualizations revealed a consistent model focus on the periocular region in medicated eyes, suggesting the detection of subtle, possibly subclinical changes such as PAP. These findings highlight the potential of artificial intelligence not only to identify medication-induced anatomical alterations invisible to the human eye but also to serve as a non-invasive tool for monitoring treatment effects.

## Supplementary Material

Supplement 1

Supplement 2
