# Supplementary material for: A Deep Learning Model for Detecting the Eyes Receiving Glaucoma Medications Using Anterior Segment Images
Source: Transl Vis Sci Technol. 2025 Aug 20;14(8):28. doi: 10.1167/tvst.14.8.28 (PMC12372948; doi:10.1167/tvst.14.8.28)

Supplementary Figure S1. False negative images identified using the deep learning model.

Thirteen of the 100 images were found to be false negatives after the machine learning model classification.

Supplementary Figure S1

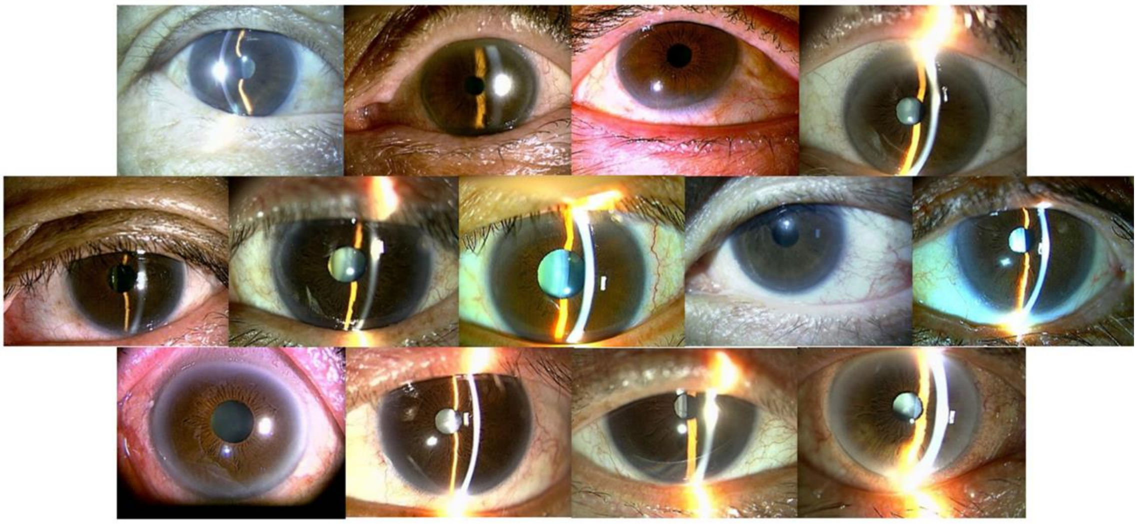

Supplementary Figure S2. False positive images identified using the deep learning model.

Eight of the 100 images were found to be false positives after the machine learning model classification.

Supplementary Figure S2

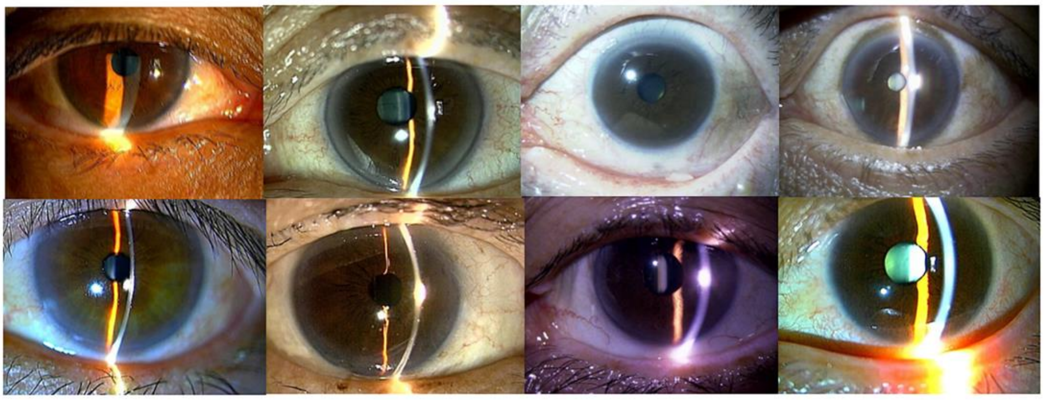

Supplement: Supplement 1 [file tvst-14-8-28_s001.pdf]
