# Supplementary material for: A Deep Learning Model for Detecting the Eyes Receiving Glaucoma Medications Using Anterior Segment Images
Source: Transl Vis Sci Technol. 2025 Aug 20;14(8):28. doi: 10.1167/tvst.14.8.28 (PMC12372948; doi:10.1167/tvst.14.8.28)
Supplement: Supplement 2 [file tvst-14-8-28_s002.pdf]

Supplementary Table S1. General Design Framework of the Deep Learning Model

Layer (type)	Output Shape	Param #
input_1 (Input Layer)	[(None, 224, 224, 3)]	0
block1_conv1 (Conv2D)	(None, 224, 224, 64)	1792
block1_conv2 (Conv2D)	(None, 224, 224, 64)	36928
block1_pool (MaxPooling2D)	(None, 112, 112, 64)	0
block2_conv1 (Conv2D)	(None, 112, 112, 128)	73856
block2_conv2 (Conv2D)	(None, 112, 112, 128)	147584
block2_pool (MaxPooling2D)	(None, 56, 56, 128)	0
block3_conv1 (Conv2D)	(None, 56, 56, 256)	295168
block3_conv2 (Conv2D)	(None, 56, 56, 256)	590080
block3_conv3 (Conv2D)	(None, 56, 56, 256)	590080
block3_pool (MaxPooling2D)	(None, 28, 28, 256)	0
block4_conv1 (Conv2D)	(None, 28, 28, 512)	1180160
block4_conv2 (Conv2D)	(None, 28, 28, 512)	2359808
block4_conv3 (Conv2D)	(None, 28, 28, 512)	2359808

block4_pool (MaxPooling2D)	(None, 14, 14, 512)	0
block5_conv1 (Conv2D)	(None, 14, 14, 512)	2359808
block5_conv2 (Conv2D)	(None, 14, 14, 512)	2359808
block5_conv3 (Conv2D)	(None, 14, 14, 512)	2359808
block5_pool (MaxPooling2D)	(None, 7, 7, 512)	0
flatten (Flatten)	(None, 25088)	0
Dense (Dense)	(None, 2)	50178

===

Total params: 14764866

Trainable params: 7129602

Non-trainable params: 7635264

Supplementally Table S2. Baseline Characteristics in the Training Dataset

	Receiving glaucoma medication	Not receiving glaucoma medication	*p*
Number	575	425	
Age, years	72.5 ± 10.1	71.2 ± 11.0	0.06
Sex			0.48
Male, n (%)	310 (53.9)	219 (51.5)	
Female, n (%)	265 (46.1)	206 (48.5)	
Eye			0.20
Right, n (%)	298 (51.8)	202 (47.5)	
Left, n (%)	277 (48.2)	223 (52.5)	
Number of glaucoma medications (n)	2.4±1.1	0	< 0.01*
Lens status			0.39
Phakic, n (%)	319 (55.5)	227 (53.4)	
IOL, n (%)	256 (44.5)	198 (46.6)	
Conjunctival hyperemia, n (%)	378 (65.7)	155 (36.5)	< 0.01*
Blepharitis, n (%)	122 (21.2)	47 (11.1)	< 0.01
Superficial punctual keratitis, n (%)	97 (16.9)	38 (8.9)	< 0.01

Abbreviations: n, number; IOL, intra ocular lens.

* Statistically significant ($p < 0.05$).

† Continuous data are presented as mean ± standard deviation.

‡ Categorical data are presented as numbers and percentages.

§ Unpaired t-tests, Pearson's chi-square test, and Fisher’s exact test were used for the analyses.

Supplementary Table S3. Type and Rate of Eye Drops in the Images of Eyes Receiving Glaucoma Medications

Type	Rate
Prostaglandin analogue, n (%)	87, (87.0)
β-blocker, n (%)	71, (71.0)
Carbonic anhydrase inhibitor, n (%)	50, (50.0)
Ripasudil, n (%)	39, (39.0)
Brimonidine, n (%)	23, (23.0)

Abbreviations: n, number

Supplementary Table S4. Glaucoma Medications in the Cases Identified as False Negatives Using the Deep Learning Model

False negative cases	Content
Case 1	Latanoprost
Case 2	Latanoprost, dorzolamide hydrochloride/timolol maleate
Case 3	Carteolol hydrochloride/latanoprost, brimonidine tartrate/ brinzolamide
Case 4	Carteolol hydrochloride
Case 5	Tafluprost, brimonidine tartrate/brinzolamide
Case 6	Omidenebag isopropyl, dorzolamide hydrochloride, timolol maleate
Case 7	Timolol Maleate
Case 8	Tafluprost/timolol maleate, brimonidine tartrate/ brinzolamide
Case 9	Timolol maleate
Case 10	Carteolol hydrochloride
Case 11	Carteolol hydrochloride/latanoprost, dorzolamide hydrochloride
Case 12	Carteolol hydrochloride/latanoprost, brimonidine tartrate/ brinzolamide
Case 13	Carteolol hydrochloride

Supplementary Table S5. Baseline Characteristics of Presence and Absence of Conjunctival Hyperemia in Section 2

	Presence of conjunctival hyperemia			Absence of conjunctival hyperemia		
	Receiving glaucoma medication	Not receiving glaucoma medication	*p*	Receiving glaucoma medication	Not receiving glaucoma medication	*p*
Number	64	31		36	69	
Age, years	70.4±10.2	69.5±8.3	0.63	69.8±12.0	68.6±12.0	0.82
Sex			0.55			0.44
Male, n (%)	33 (51.6)	18 (58.1)		18 (50.0)	29 (42.0)	
Female, n (%)	31 (48.4)	13 (41.9)		18 (50.0)	40 (58.0)	
Eye			0.23			0.62
Right, n (%)	33 (51.6)	20 (64.5)		18 (50.0)	38 (55.1)	
Left, n (%)	31 (48.4)	11 (35.5)		18 (50.0)	31 (44.9)	
Number of glaucoma medications (n)	2.9±1.4	0	< 0.01*	2.4±1.3	0	< 0.01*
Lens status			0.78			0.47
Phakic, n (%)	35 (54.7)	16 (51.6)		22 (61.1)	47 (68.1)	

IOL, n (%)	29 (45.3)	15 (48.4)		14 (38.9)	22 (31.9)	
Glaucoma, n (%)	62 (96.9)	10 (32.2)	< 0.01*	33 (91.7)	9 (13.0)	< 0.01*

Abbreviations: n, number; IOL, intraocular lens

* Statistically significant ($p < 0.05$).

†Continuous data are presented as mean ± standard deviation.

‡Categorical data are presented as numbers and percentages.

§Unpaired t-tests and Pearson's chi-square test were used for the analyses.

Supplementary Table S6. Baseline Characteristics of Presence and Absence of Prostaglandin Analogue Eye Drops in Section 3

	Presence of prostaglandin analogue			Absence of prostaglandin analogue		
	Receiving glaucoma medication	Not receiving glaucoma medication	*p*	Receiving glaucoma medication	Not receiving glaucoma medication	*p*
Number	87	100		13	100	
Age, years	69.8±10.2	68.9±11.0	0.86	71.6±14.8	68.9±11.0	0.36
Sex			0.44			0.99
Male, n (%)	45 (51.7)	46 (46.0)		6 (46.2)	46 (46.0)	
Female, n (%)	42 (48.3)	54 (54.0)		7 (53.8)	54 (54.0)	
Eye			0.59			0.42
Right, n (%)	47 (54.0)	58 (58.0)		6 (46.2)	58 (58.0)	
Left, n (%)	40 (46.0)	42 (42.0)		7 (53.8)	42 (42.0)	
Number of glaucoma medications (n)	3.0±1.3	0	< 0.01*	1.2±0.4	0	< 0.01*
Lens status			0.44			0.52
Phakic, n (%)	50 (57.5)	63 (63.0)		7 (61.1)	63 (63.0)	
IOL, n (%)	37 (42.5)	37 (37.0)		6 (38.9)	37 (37.0)	
Glaucoma, n (%)	83 (95.4)	19 (19.0)	< 0.01*	12	19 (19.0)	< 0.01*
Conjunctival	60 (69.0)	31 (31.0)	< 0.0	4 (30.8)	31 (31.0)	1.0

hyperemia, (%)			1*		
Grade, n					
Grade 1	22	20		2	20
Grade 2	28	10		2	20
Grade 3	10	1		0	1

Abbreviations: n, number; IOL, intra ocular lens

* Statistically significant ($p < 0.05$).

†Continuous data are presented as mean ± standard deviation.

‡Categorical data are presented as numbers and percentages.

§Unpaired t-tests, Pearson's chi-square test, and Fisher’s exact test were used for analyses.
